# Audit on Venous Thromboembolism (VTE) Assessment in Adult Inpatient Wards on Admission

**DOI:** 10.1192/bjo.2025.10668

**Published:** 2025-06-20

**Authors:** Syeda Huma Shah

**Affiliations:** Southwest Yorkshire Partnership NHS Foundation Trust, Halifax, United Kingdom

## Abstract

**Aims:** Aim of this audit was to assess if VTE (Venous thromboembolism) assessments on admission to adult inpatient wards (two working age and one old age ward) at the Inpatient Psychiatry unit are carried out as per the local Trust’s Policy.

**Methods:** I made a questionnaire comprising 6 questions, based on the local Trust’s VTE assessment policy.

Data was reviewed for a total of 70 patients but collected for 54 patients between 17/04/2024 and 07/05/2024 admitted on all three wards at the Acute Inpatient Psychiatric Unit.

16 patients were excluded due to them being transfer patients from other units and not new admissions.

Data was collected from patients’ electronic records which included VTE assessment risk forms, progress notes, initial psychiatric assessment forms on admission, physical examination forms and Multidisciplinary team reviews.

Data entry and analysis was done using Microsoft Word and Excel.

**Results:** Based upon the Trust’s policy, the following practices/guidelines were checked for compliance against the expected standard:

1. Was the VTE assessment carried out on admission? Standard – 100%. Compliance – 68%.

2. Was the VTE assessment questionnaire completed correctly as per Trust’s Policy on patient’s electronic record system? Standard – 100%. Compliance – 66.6%.

3. Were the VTE related Examination findings documented in the Physical Examination section/form on the Electronic Record System? Standard – 100%. Compliance – 63.4%.

4. Was VTE risk re-assessed on consultant review? Standard – 100%. Compliance – 1.8%.

5. Were the patients assessed to be ‘at risk’ of developing VTE, re-assessed within 24 hours of admission or later if the patient’s condition changed? Standard – 100%. Compliance – 33.3%.

6. Were all patients assessed to be at risk of VTE offered thromboprophylaxis that is consistent with NICE and Trust guidelines unless contraindicated? Standard – 100%. Compliance – 100%.

**Conclusion:** Results showed that the current practice standards are below the expected standard in all areas except prescribing the correct prophylactic medication if indicated. Based on these results, the following recommendations were made:

Incorporate detailed information about the Trust’s local VTE assessment policy into resident doctor’s induction.

Duty doctors to handover incomplete assessments to ward team doctors to avoid missing the assessment in case patients refuse it on admission/unable to carry out for some reason.

Informative poster to be pasted on the board at Resident Doctor’s room to reinforce the practice.

Pop-up/Prompts are already being given every time on opening patient’s Electronic Records for incomplete VTE assessments. Duty doctors to please complete them as soon as possible if prompted.

Consultants to discuss about frequency of VTE assessments on MDT reviews during discussion at the end of the audit presentation.

Repeat second cycle of audit after 6–8 months.

